# Automatic classification of healthy and disease conditions from images or digital standard 12-lead electrocardiograms

**DOI:** 10.1038/s41598-020-73060-w

**Published:** 2020-10-01

**Authors:** Vadim Gliner, Noam Keidar, Vladimir Makarov, Arutyun I. Avetisyan, Assaf Schuster, Yael Yaniv

**Affiliations:** 1 Computer Science Department, Technion-IIT, Haifa, Israel; 2 Laboratory of Bioenergetic and Bioelectric Systems, Biomedical Engineering Faculty, Technion-IIT, Haifa, Israel; 3 System Programming Lab, Novgorod State University, Veliky Novgorod, Russia; 4 Ivannikov Institute for System Programming of the Russian Academy of Sciences, Moscow, Russia

**Keywords:** Cardiology, Machine learning

## Abstract

Standard 12-lead electrocardiography (ECG) is used as the primary clinical tool to diagnose changes in heart function. The value of automated 12-lead ECG diagnostic approaches lies in their ability to screen the general population and to provide a second opinion for doctors. Yet, the clinical utility of automated ECG interpretations remains limited. We introduce a two-way approach to an automated cardiac disease identification system using standard digital or image 12-lead ECG recordings. Two different network architectures, one trained using digital signals (CNN-dig) and one trained using images (CNN-ima), were generated. An open-source dataset of 41,830 classified standard ECG recordings from patients and volunteers was generated. CNN-ima was trained to identify atrial fibrillation (AF) using 12-lead ECG digital signals and images that were also transformed to mimic mobile device camera-acquired ECG plot snapshots. CNN-dig accurately (92.9–100%) identified every possible combination of the eight most-common cardiac conditions. Both CNN-dig and CNN-ima accurately (98%) detected AF from standard 12-lead ECG digital signals and images, respectively. Similar classification accuracy was achieved with images containing smartphone camera acquisition artifacts. Automated detection of cardiac conditions in standard digital or image 12-lead ECG signals is feasible and may improve current diagnostic methods.

## Introduction

Because of its simplicity, noninvasiveness and low cost, standard 12-lead electrocardiography (ECG) is used as the primary clinical tool to diagnose changes in heart conditions. It has been shown as the single adequate source to diagnose cardiac rhythm^1^ and morphology^2^, such as cardiac arrhythmia^3^, acute and prior myocardial infarctions^4^, pericardial disease^5^ and atrial or ventricular enlargement^6^.

The value of automatic 12-lead ECG diagnostic approaches lies in their ability to screen the general population and to provide an additional opinion for health care providers. Since 1957, attempts have been made to automate interpretation of ECG recordings, with emphasis on atrial fibrillation (AF)-related findings. Yet, currently available automated algorithms have demonstrated mediocre performance^7^. Thus, despite current technologies, particularly in the areas of advanced machine learning and artificial intelligence (AI) methods, the clinical utility of automated ECG interpretations remains limited and standard 12-lead ECG recordings are still evaluated by cardiologists. There are several challenges that embrace the design of clinically relevant automated algorithms of classification of cardiac conditions from 12-lead ECGs. Achieving high accuracy is the first step. Note that even experienced cardiologists can disagree, thus achieving high accuracy is challenging even for cardiologists^8^. In recent years, advanced machine learning and AI methods have been applied to classify one-lead ECGs^2,9–11^ and have provide sensitivity to most rhythm classes slightly exceeding that of the average cardiologist. These methods comprise a deep genetic ensemble of classifiers^12,13^, attention mechanisms on ECG strip^14^, feature extraction and voting methods^15^, different pattern recognitions based on transforms, e.g., wavelet transform^16^, unique feature utilization, e.g., output of Welch transform^17^, discrete Fourier transform^17^, spectral power density transform^17^ and evolutionary neural systems^18^. However, to date, all these methods have only been applied on single-lead ECG, and therefore are only suitable for classification of select diseases, which are mainly rhythm conditions. Translation of such algorithms to 12-lead ECG recordings, which enable detection of both rhythm and morphology conditions, is complicated by the format of available databases which are sometimes available in digital signal format and sometimes as machine plot images. Thus, a general, hybrid approach that allows for disease identification from both types must be developed. Moreover, because machine plot images are now scanned by mobile devices, the approach must take into account artifacts introduced by mobile device image acquisition. In addition, standardization of the applied algorithms requires a large dataset at the development stage. However, to the best of our knowledge, a database with standard, open-source, 12-lead ECG records from a heterogenous group of patients and healthy volunteers, that includes both digital and machine plot formats, does not exist. Apart from the aforementioned technical hurdles, multiple disorders are often concomitantly present in the same ECG record. However, the majority of the published methods classify the ECG into a single disease category^3,19,20^. In addition, as an AI system must be trained on labeled data, there will always be disorders which do not appear in a specific training dataset, questioning the applicability of such algorithms to these additional diseases. Thus, only a generic architecture that can be applied to many disease types and which can detect all disorders appearing in the same record, will be of physiological relevance.

This work aimed to develop a two-way approach to a standard 12-lead ECG database, that can use as an input either standard digital or image 12-lead ECG signals and would classify recordings as normal sinus or one or more of the following disorder types: atrial fibrillation (AF), first-degree atrioventricular block (I-AVB), left bundle branch block (LBBB), right bundle branch block (RBBB), premature atrial contraction (PAC), premature ventricular contraction (PVC), ST-segment depression (STD) or ST-segment elevation (STE). There are several innovative aspects that are included in this paper: (1) This proof-of-concept work demonstrated that an automated ECG interpretation system can be created and can reach high accuracy using deep learning tools. (2) An open-source database, which is accessible upon request and is compatible with a well-known and frequently used deep learning framework (PyTorch), was established. (3) The work developed a generic deep network architecture that can be applied to many diseases of different types with high accuracy, can detect all disorders appearing in the same record, and can be extended to additional diseases with relatively minimal effort. (4) The deep network architecture proved compatible with both digital 12-lead signals (i.e., 13 signals) and with standard plots of all leads, which are typically printed on paper. (5) The deep network architecture proved compatible with images with background noise and change of plot perspective that observed when ECG plot is captured by mobile device. Achieving these innovative aspects will promote the generation of an automated 12-lead ECG diagnostic system that will allow for screening of the general population in any clinic equipped with a standard 12-lead ECG machine and provide a second opinion for the health care provider.

## Methods

The database, analytic methods, and study materials that support the findings of this study are available upon request.

### Data source and standard 12-lead ECG database generation

**Figure 1:**
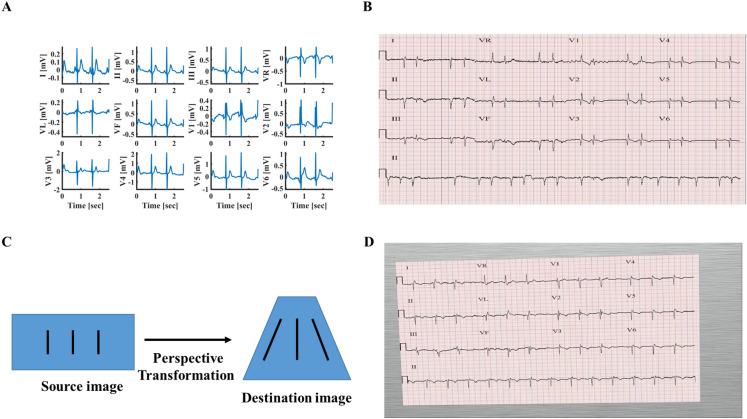
Examples of data format. (**A**) Digital 12-lead ECG signal data. (**B**) A rendered ECG image. The digital signal was plotted on a publicly available ECG paper template^29^. The lead labels indicate lead recording locations on the template (2.5 s each). The lower long lead recording is lead II (10 s). The calibration signal is also drawn on the left side of each row. (**C**) Image without (left) and with (right) perspective transformation. The perspective transformation imitates human (or camera) view of a rectangular ECG plot. (**D**) Rendered image after applying a random perspective transformation and a random background to simulate acquisition by a mobile device, such as a smartphone.

The database was constructed using 6877 published digital (Fig. 1A) patient records (female 3178; male 3699), of durations of 6–60 s, that were collected in 11 hospitals^21^. ECG recordings were acquired at a sampling rate of 500 Hz. The data were classified by board-certified practicing cardiologists in these 11 hospitals as normal sinus or one or more of the following conditions: AF, I-AVB, LBBB, RBBB, PAC, PVC, STD or STE. Because standard 12-lead ECG records consist of of 2.5-s recording for each one of the 12 leads and 10-s recordings for lead II, recordings longer than 10 s were divided to 10-s segments with no overlaps. Each 10 s segment was used to generate four standard ECG records by taking 2.5 s of 12 leads without overlap, and the same 10 s of lead II. The 11 ECG records shorter than 10 s were omitted. Following these steps, a database of 41,830 recordings (in hdf5 format^22^ compatible with PyTorch) from 6866 patients (female 3174; male 3692) was created. The dataset scheme followed that of other known databases (e.g., MNIST). A “one-hot” classification binary vector with a length of 9 was generated for each database entry: (1) Normal, (2) AF, (3) I-AVB, (4) LBBB, (5) RBBB, (6) PAC, (7) PVC, (8) STD and (9) STE. The corresponding vector entry was “1” if the disease existed in the database entry and otherwise was “0”. Table 1 presents the number of patients and records in each category.

**Table 1:** Classification of source data, with number of patients and records for each condition type.

Condition type	# of patients	# of records
Normal	918	5453
AF	1220	7080
I-AVB	721	3969
LBBB	235	1356
RBBB	1853	10,401
PAC	614	4552
PVC	699	5598
STD	868	5069
STE	220	1458

*AF* Atrial fibrillation, *I-AVB* first-degree atrioventricular block, *LBBB* left bundle branch block, *RBBB* right bundle branch block, *PAC* premature atrial contraction, *PVC* premature ventricular contraction, *STD* ST-segment depression, *STE* ST-segment elevation.

### The image dataset

ECG recordings are available in two modalities—digital signal or paper plots. Our goal was to show that automated identification of diseases can be achieved with standard available ECG data, regardless of their format. Because there is no accessible dataset of labeled ECG plots with a sufficiently large volume of data, we generated such a set from the digital signal dataset^21^.

A publicly available template of ECG paper was used to generate ECG images (see Fig. 1B). Of note, the machine learning techniques we used were agnostic to the specific ECG paper template, thus identical results can be obtained using any template. Each small red box represents 0.04 s (imitating ECG paper that is fed into the machine at a rate of 25 mm/s) and 1-mm height. ECG lead label was added next to each lead plot and a calibrated signal (10 mm-high and 0.2 s-wide) was added at the beginning of each ECG line.

When the data are available in paper plot form only, the easiest way to make it accessible to an automated classification system is by capturing the plot image, which then serves as the input to the system. Today, it is easy to capture the plot image using cameras in smartphones or other mobile devices. However, this practice is likely to incorporate distortions in the resulting image. Because of lack of an accessible database comprising labeled ECG images captured by smartphones, we simulated the artifacts caused by smartphone acquisition of ECG images, by applying random perspective image transformations to imitate projection from 3D view into 2D image (Fig. 1C) and added random backgrounds resembling various table textures (Fig. 1D).

### Data sets for developing and testing the neural network

After an initial split of the data into development (83% of the population; n = 36,000 records) and holdout (17% of the population; n = 5830 records) datasets, the development dataset was further divided into training (95% of the development set) and internal validation (5%) datasets. To avoid cross-contamination, the development and holdout datasets consisted of data from different sets of patients. Similarly, the training and test sets consisted of different sets of patients. Training both the deep convolution neural network (CNN) for digital data (CNN-dig) and the CNN for plot images (CNN-ima) networks, was done using the Adam^23^ optimizer with binary cross-entropy as the loss function. The internal validation data set was used to tune the hyper-parameters and to select the optimal model. The same data split (training, validation, and testing sets) was used for both the digital signal and image networks.

### Overview of the deep network model

**Figure 2:**
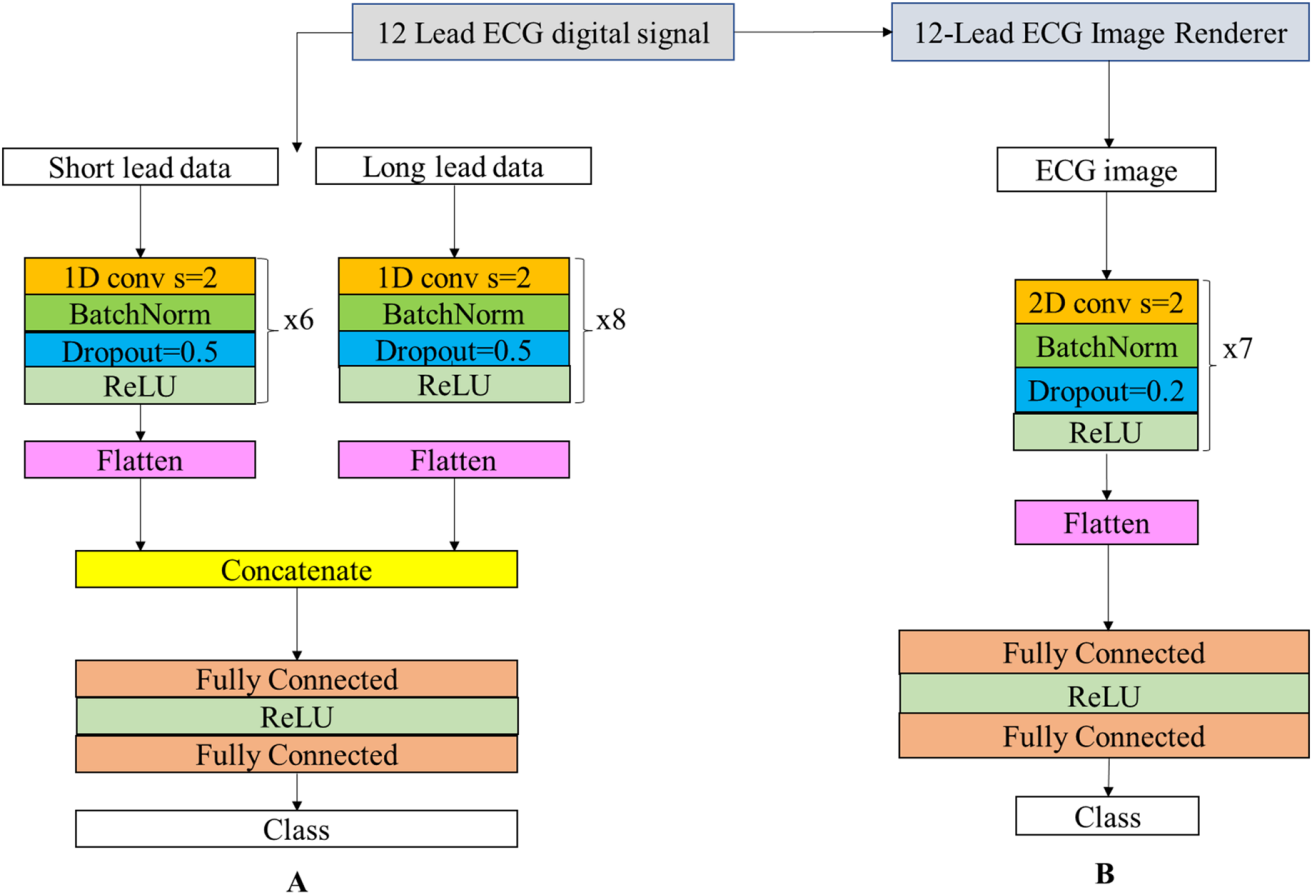
CNN architectures. (**A**) 12-lead ECG digital signal data classification net CNN-dig, and (**B**) 12-lead ECG image classification net CNN-ima. In both networks, the input enters a convolution layer with a stride step of 2. Next is a batch normalization^30^ layer in which the batch distribution is normalized. The dropout^31^ layer randomly deletes a fraction of the network edges with a given probability during the training to improve robustness. Next, the 3D convolution output is flattened, goes through a fully connected classification layer and serves as a thresholder for the final outcome. Notice that each of the resulting networks is trained on a single disease and generates a binary output for each input: in class or out of class. Kernel size is 17 for digital signal processing net, and 7 × 7 for image processing net.

CNNs were implemented using the Pytorch Framework with Python. The same basic architecture was used in different variations for all digital signal identification networks (CNN-dig) (Fig. 2A) and for all image identification networks (CNN-ima) (Fig. 2B). CNN-ima takes color images (RGB) of size of 675 pixels on 1450 pixels as an input, thus forming a tensor of [675,1450,3].

While CNNs are mainly applied to images, we adjusted the network architecture and the convolution kernel size to produce spatial and temporal feature extraction layers. The network was trained by adjusting the weights of the convolutional filters to extract meaningful and relevant features in an unsupervised way. Both the ECG image and digital signal networks were built using stacked blocks of convolutional, batch normalization and dropout layers (Fig. 2). For training with digital signal data, two separate stacked blocks were used to extract temporal features from short leads and from the long lead. After flattening each stacked block (either short or long lead), the features extracted from both blocks were concatenated and were used as input to a fully connected network. Both networks used a linear rectifier (ReLU) as their activation function. The difference between the image CNN-dig and digital signal CNN-ima networks was in the convolution output. The image network is one stack of convolutional layers followed by a fully connected layer (see Fig. 2A). Another difference is the 1D convolution kernels in the digital signal network (17) versus 2D convolution kernels in the image network (7 × 7). Hyperparameters (batch size, initial learning rate, number of nodes in the fully connected layers, and number of convolutional layers) were adjusted during training to obtain the optimal model. Initial learning rates were set in the range of 1e–2 to 1e–5, and testing batch sizes varied in the range of 50–150 for the image network CNN-ima, and 150–256 for the digital signal network CNN-dig. The actual used learning rate was selected in such a way that the learning process would be effective, i.e. large enough to converge with reasonable speed, on the one hand, but small enough to sustain the learning process and avoid divergent behaviors. We tested different learning rates for several epochs to find the one suitable for our needs. With respect to convolution kernel, we tested a span of convolution kernels, plotted the performance versus convolution kernel size, fitted a parabolic function and chose the kernel which provided optimal accuracy. Selected hyperparameters included batch size of 256 for the digital signal network, whereas for the image network, we chose a batch size of 150. Note that for the digital signal case, the same net architecture (Fig. 2A) was used for each condition (8 diseases and normal sinus), while, obviously, the model parameters (the outcome of the training process) differed. Optimal CNN structure and parameters are described in the legend of Fig. 2.

Network depth was optimized in such way that, on the one hand, the number of trainable parameters will fit the size of the training dataset, but on the other hand, the net will be deep enough to learn complex features and provide high detection accuracy. We tried different numbers of staggered convolution blocks (5–8; see Fig. 2) both for short-lead data and long-lead data, and eventually used 6 staggered blocks for short-lead data and 8 for long-lead data for CNN-dig, because it provided the best accuracy. With regards to CNN-ima, we tested the same span of blocks (5–8) and selected 7 staggered convolution layers because it provided the best results. With respect to Adam optimizer learning rates, we tested a set of learning rates [10–3,10–4,10–5,10–6] and selected the one which allowed the learning process to converge at a reasonable rate (10^–4^).

### Training process

Deep net implementation was performed in Python, using the PyTorch framework (version 1.4.0). In addition, torchvision (version 0.5.0), numpy (version 1.17.5) and openCV (version 4.1.1) libraries were used.

The server used for training was: Intel(R) Core(TM) i9-7900X @ 3.30 GHz. RAM: 32 GB. GPU: GeForce GTX 1080 Ti (of NVIDIA). Cuda version was 10.2. OS: Linux version 4.15.0-108-generic.

Training time of CNN-dig was 20 s per epoch, whereas a maximum of 100 epochs was defined (unless early stopping condition was reached, i.e., 3 epochs in which training loss is getting smaller but test loss is not). With regards to CNN-ima, training time was 9 min and 20 s per epoch, and after 9 epochs, the stop condition was reached. With respect to classification, CNN-ima classified one records in about 2 s, whereas CNN-dig did so in the sub-second period.

### Model evaluations and statistical methods

For each disease, a binary classifier (disease exists or not) was designed with an output P, where P was in the range of 0–1. In cases of inconclusive discrimination, P will be closer to 0.5, thus, a trivial threshold of 0.5 was used for final binary classification. In the training stage, the networks were fed the entire training set. After every iteration (epoch), the model was evaluated on the validation set. The software callback saved only the best model based on the validation performance (checkpoints). To avoid overfitting, we used the early stopping method. Training was terminated when the validation performance ceased to improve over 5 or 10 consecutive epochs, for CNN-ima or CNN-dig, respectively. Our experience showed that CNN-dig had to be trained for 100 epochs, whereas the CNN-ima achieved optimal results already after 6 epochs.

The following metrics were used to assess performance of the networks (True positive (TP), True negative (TN), False positive (FP), False negative (FN), Accuracy (ACC), Sensitivity (TPR), Specificity (TNR), Positive prediction value (PPV), Negative predictive value (NPV)):

1.1 $$ TP = \frac{{\# \,correctly\;detected\;disease\;occurences{ }}}{\# \,episodes\;with\;presence\;of\;the\;disease} $$

1.2 $$ TN = \frac{{\# \,correctly\;detected\;disease\;absence}}{{\# \,episodes\;with\;absence\;of\;the\;disease}} $$

1.3 $$ FN = \frac{{\#\,misdetected\;disease\;occurences}}{{\# \,episodes\;with\;presence\;of\;the\;disease}} $$

1.4 $$ FP = \frac{{\# \,incorectly\;classified\;episodes}}{\# \,episodes\;with\;absence\;of\;the\;disease} $$

1.5 $$ Sensitivity{:}\;True\;positive\;rate \left( {TPR} \right) = \frac{TP}{{TP + FN}} $$

1.6 $$ Specificity{:}\;True\; negative\, rate\; \left( {TNR} \right) = \frac{TN}{{TN + FP}} $$

1.7 $$ Precision\;or\;positive\;predictive\;value{:}\;PPV = \frac{TP}{{TP + FP}} $$

1.8 $$ Negative\;predictive\;value{:}\;NPV = \frac{TN}{{TN + FN}} $$

1.9 $$ Accuracy{:}\;ACC = \frac{TP + TN}{{TP + TN + FP + FN}} $$

1.10 $$ F_{1} = \frac{2*TP}{{2*TP + FP + FN}} $$

## Results

### Creation of a relatively large, publicly available, standard12-lead ECG database

A database of 41,830 standard 12-lead ECG records collected from a total of 6866 patients and volunteers, was created. Each digital ECG recording was classified to one or more of nine categories (see “Methods” section). In total, there were 13.03% normal, 16.92% AF, 9.48% I-AVB, 3.24% LBBB, 24.86% RBBB, 10.88% PAC, 13.38% PVC, 12.11% STD and 3.48% STE recordings. The training set, including the validation set, was comprised of 36,000 ECG recordings. The testing set was comprised of 5830 ECG recordings collected from individuals who were not included in the training set.

### Generic, optimal convolution kernel size for digital signal data

To identify a network architecture suitable for as many other diseases as possible, including both morphological and rhythm disorders, we searched for the optimal convolution kernel size providing high accuracy for all conditions. Small convolution kernels are expected to be more suitable for identifying morphological disorders, while large convolution kernels are expected to be sensitive to changes between subsequent beats and therefore suitable for identifying both rhythm and morphological disorders. Table 2 shows that a kernel size of 17 provided the best average performance over all disease types.

**Table 2:**
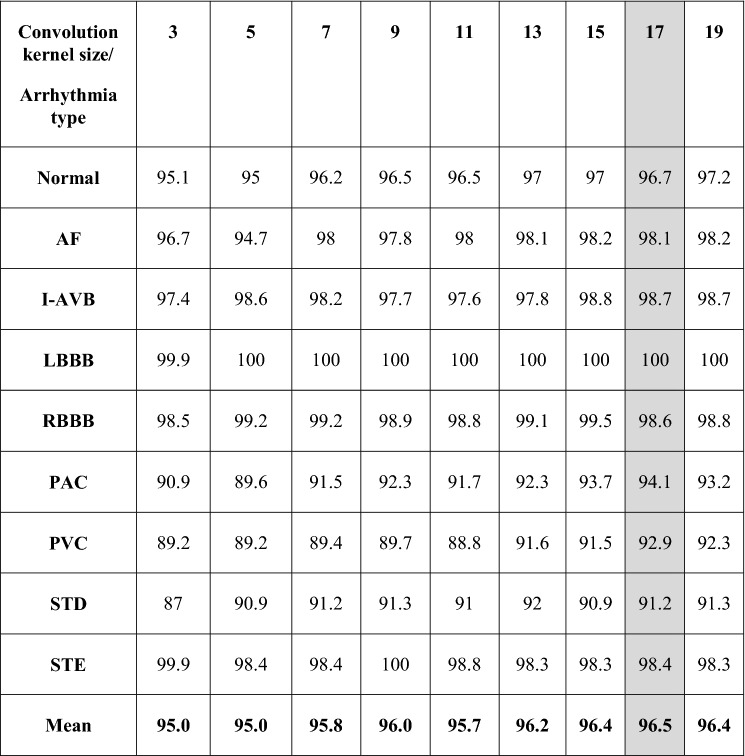
Convolution kernel size optimization using the validation set (1000 records).

*AF* Atrial fibrillation, *I-AVB* first-degree atrioventricular block, *LBBB* left bundle branch block, *RBBB* right bundle branch block, *PAC* premature atrial contraction, *PVC* premature ventricular contraction, *STD* ST-segment depression, *STE* ST-segment elevation.

### Detection of multiple disorders from digital signal data: generalizability and extensibility

**Figure 3:**
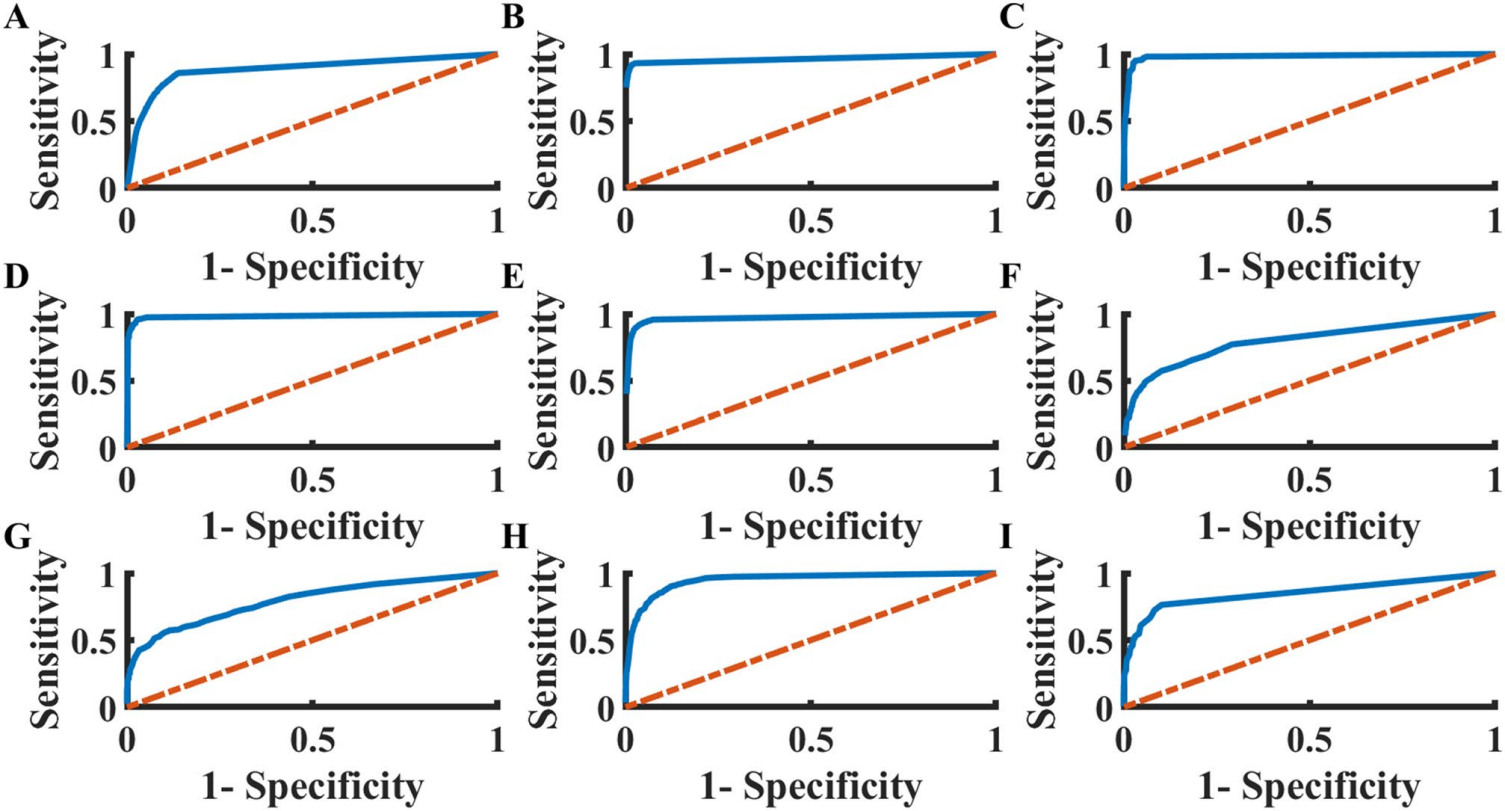
The receiver operation characteristic (ROC) of disease identification from digital signals, using CNN-dig. (**A**) Normal, area under curve 0.89, (**B**) AF, area under curve 0.96, (**A**,**C**) I-AVB, area under curve 0.98, (**D**) LBBB, area under curve 0.98, (**E**) RBBB, area under curve 0.97, (**F**) PAC, area under curve 0.80, (**G**) PVC, area under curve 0.80, (**H**) STD, area under curve 0.95, (**I**) STE, area under curve 0.85. *AF* Atrial fibrillation, *I-AVB* first-degree atrioventricular block, *LBBB* left bundle branch block, *RBBB* right bundle branch block, *PAC* premature atrial contraction, *PVC* premature ventricular contraction, *STD* ST-segment depression, *STE* ST-segment elevation.

Figure 3 illustrates the receiver operation characteristic (ROC) of each disease classification. Overall area under the curve for each disease was between 0.8 and 0.98. Table 3 shows the statistical measurements for each disease. The accuracy (in gray) was 92.9–100%, depending on the disease type.

**Table 3:**
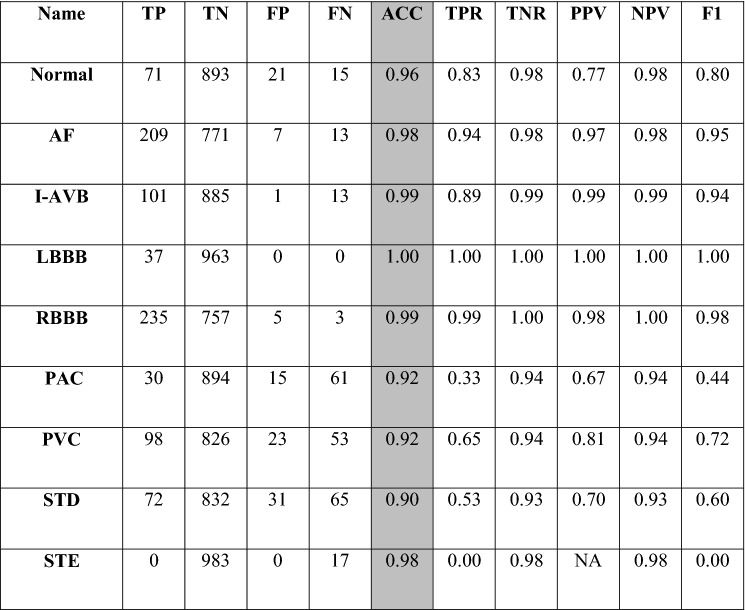
Performance statistics for each disease type, as determined using digital signals.

*AF* atrial fibrillation, *I-AVB* first-degree atrioventricular block, *LBBB* left bundle branch block, *RBBB* right bundle branch block, *PAC* premature atrial contraction, *PVC* premature ventricular contraction, *STD* ST-segment depression, *STE* ST-segment elevation, *TP* true positive, *TN* true negative, *FP* false positive, *FN* false negative, *ACC* accuracy, *TPR* sensitivity, *TNR* specificity, *PPV* positive prediction value, *NPV* negative predictive value.

**Figure 4:**
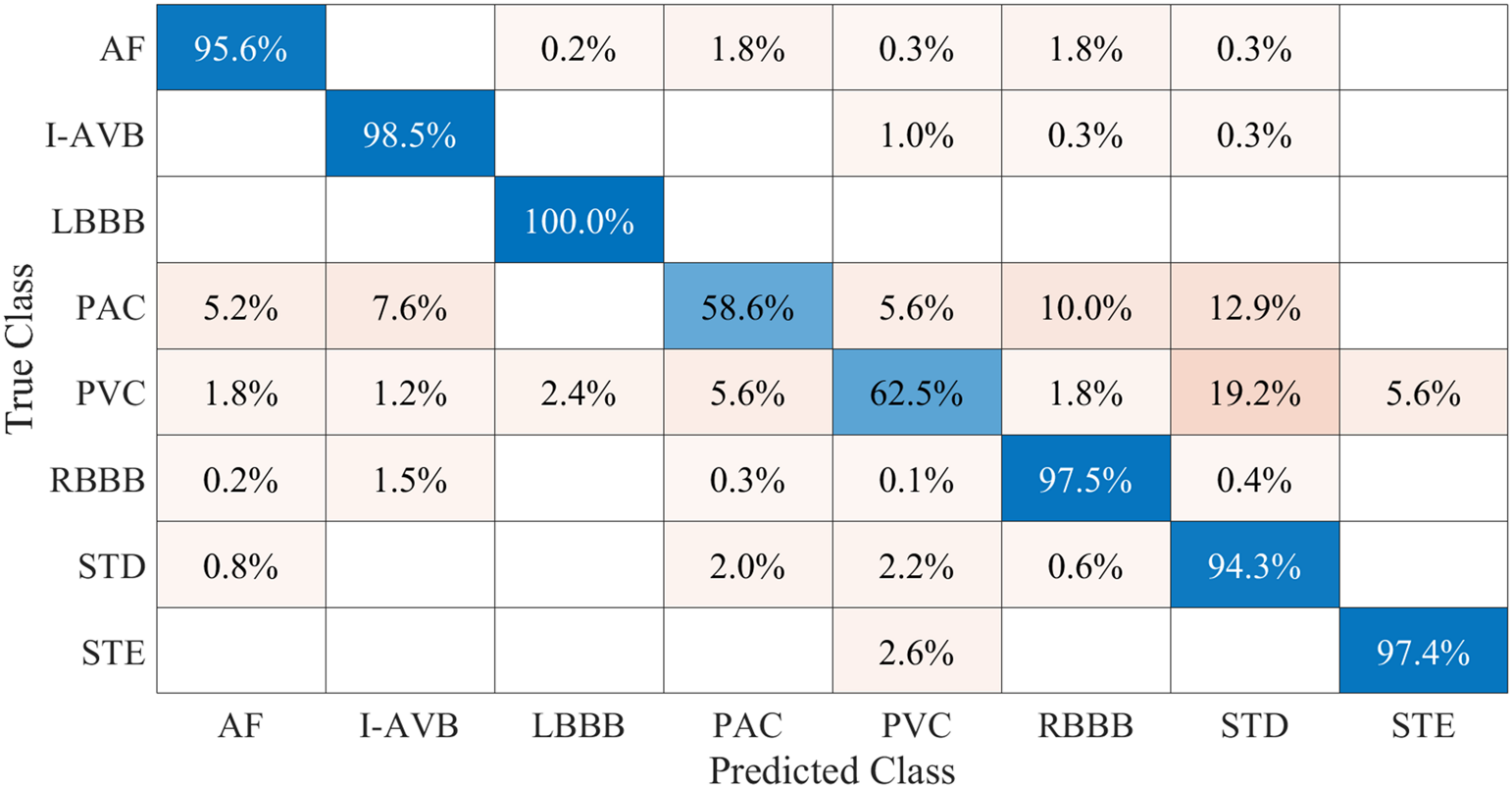
Confusion matrix of predicted class versus true class. *AF* Atrial fibrillation, *I-AVB* first-degree atrioventricular block, *LBBB* left bundle branch block, *RBBB* right bundle branch block, *PAC* premature atrial contraction, *PVC* premature ventricular contraction, *STD* ST-segment depression, *STE* ST-segment.

A confusion matrix was generated to identify trends in misidentification in our network (Fig. 4). Because our method was based on independent identification of each cardiac disorder, only data from the test set which included only one classified disease (annotated as well as predicted) were used for the confusion matrix. Out of 5830 records, 3184 were used to generate the matrix. Two significant trends of misidentification were detected (Fig. 4): PAC was mistakenly classified as either RBBB or STD and PVC was mistakenly classified as STD.

### Atrial fibrillation identification from ECG images

**Figure 5:**
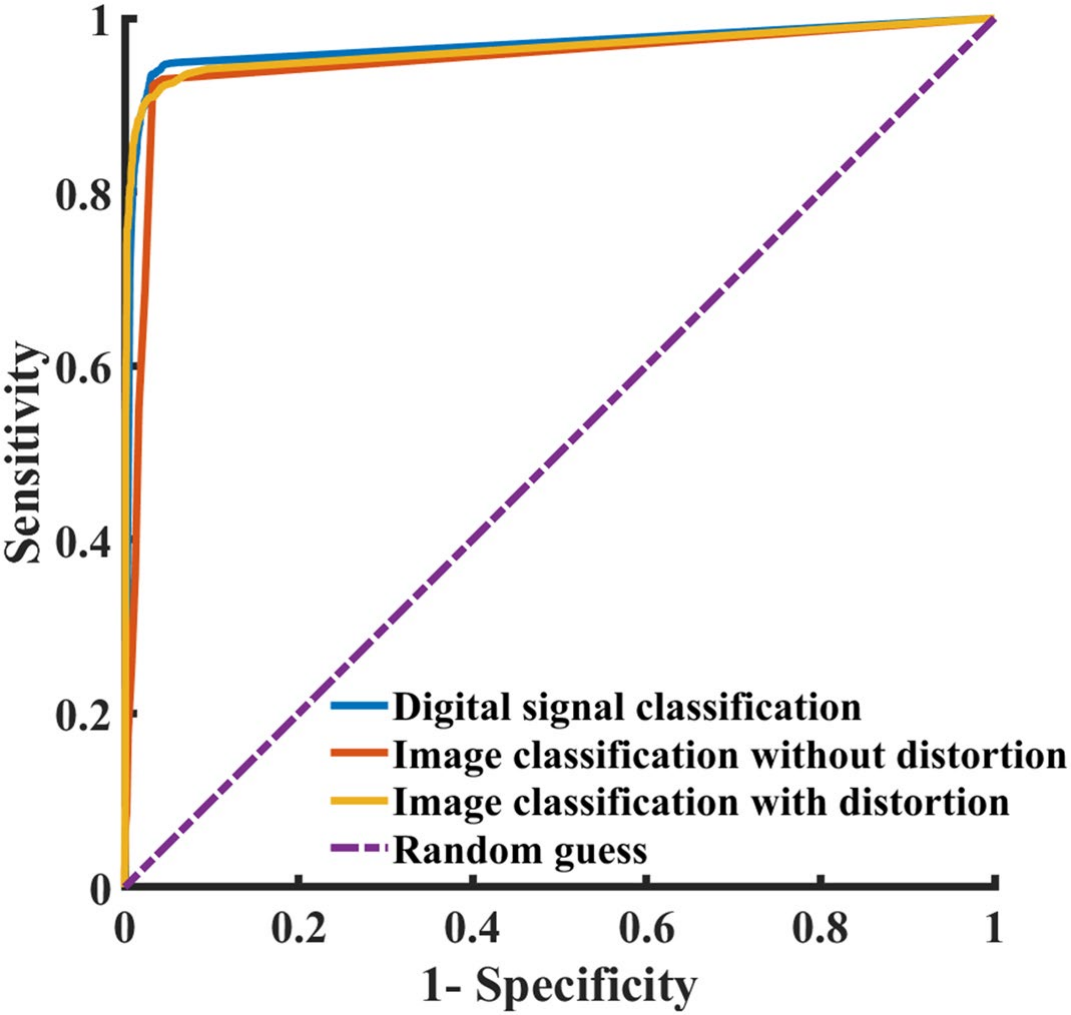
The receiver operation characteristic (ROC) of atrial fibrillation detection. ROCs of digital ECG signal classification (area under curve: 0.96), ECG image classification without distortions (area under curve: 0.94), ECG image classification with distortions (area under curve: 0.96) and random guess (area under curve: 0.5).

Because of added redundant information (e.g., red squares), it may seem that identifying diseases from 2D images is harder than doing so with 1D digital signals. This intuition was challenged by training CNN-ima to identify AF, the most common arrhythmia, using images only. Figure 5 illustrates the ROC curve for digital versus image input signals. Similar degrees of accuracy were achieved with digital (98%) as compared to image (96%) input signals.

### AF identification from ECG images with mobile device acquisition distortion

Today, images of ECG plots sent for automatic image classification, are likely to be acquired using smartphones, and therefore are likely to be distorted. Due to the lack of an adequate labelled dataset of ECG images, distorted image ECG plots were simulated by employing random perspective transformations as well as incorporating random backgrounds. Figure 5 illustrates the ROC curve for digital versus image signals, with versus without perspective transformation and random texture background. As previously shown, AF identification was slightly less accurate using ECG images as compared to digital ECG recordings. In addition, AF identification of ECG images with distortion was a bit less accurate than without.

### Classification accuracy versus sample size

**Figure 6:**
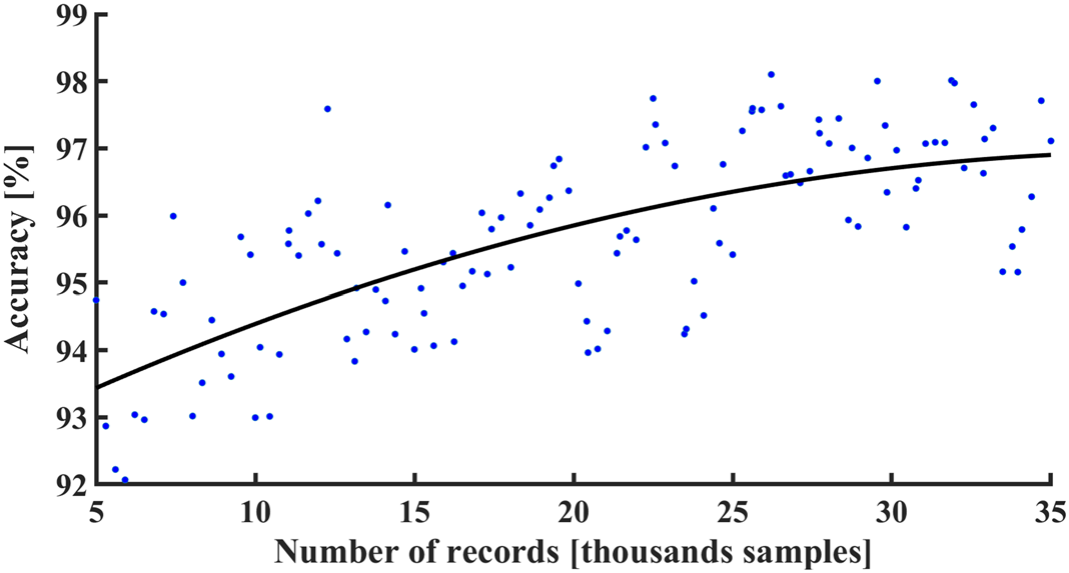
Binary accuracy. Accuracy of binary classification of atrial fibrillation as a function of training set size.

In deep learning, the output accuracy depends on the number of training samples. To determine the size-effect of our results and to quantify how additional data may improve them, we explored the dependence of AF identification accuracy on the number of training samples. To this end, the CNN-dig model was trained with increasing amounts of training data, and then tested each time with the same test set. Figure 6 shows how increasing the volume of training data increased the accuracy of the results. Moreover, the fitting curve (${\text{y}} = a \cdot {\sin}\left( {b \cdot x + c} \right)$), coefficients which were found by optimization) showed that no saturation was achieved, namely, that additional data would result in higher accuracy.

## Discussion

Standard 12-lead ECG is one of the most common tools used for cardiac disease diagnosis and is easily accessible in any clinic. This proof-of-concept work demonstrated that an automated ECG interpretation system can be created and can reach high accuracy using deep learning tools. The proposed generic deep network architecture can be applied to many conditions of different types (i.e., rhythm or morphology conditions) with high accuracy, can detect all disorders appearing in the same record, and can be extended to additional diseases with relatively minimal effort. In addition, the approach was shown to be applicable to both digital ECG and ECG image signals, including images captured by smartphones. Such an automated system would provide a second opinion on manually assessed ECG results and cost-effectively scan massive at-risk populations.

12-lead ECG is essential for accurate diagnosis of both morphological and rhythm disorders. Moreover, this device can be found at any clinic. However, interpretations are currently manually performed by clinicians, without any means of automation. AI methods provide promising new platforms for development of such automated arrhythmia detection tools. To apply these methods, a large dataset was essential. However, to the best of our knowledge, a database of standard, open-source, 12-lead ECG records from many patients and healthy volunteers does not exist. As part of this research, we built such a database, which is accessible upon request, and compatible with a well-known and frequently used deep learning framework (PyTorch). We believe that availability of this database will very positively impact future research in the field.

A multiclass model^3,19,20^ was recently suggested for automated classification of cardiac diseases. However, the straightforward learning approach proposed in these works had two critical limitations. First, more than one disorder commonly concomitantly present in the same ECG record. Second, disorders other than the one considered in the multiclass model exist, which raises the question whether published results are applicable to these additional diseases. To overcome these limitations, we proposed a generic deep network architecture that can be applied to many diseases of different types with high accuracy, can detect all disorders appearing in the same record, and can be extended to additional diseases with relatively minimal effort.

Analysis of 12-lead ECG recordings must be sufficiently sensitive to identify and differentiate between concomitant conditions. Assignment of such a task to a multiclass classifier would require training for the theoretical number of disease combinations (2^8^), making our classifier cumbersome, inefficient, and practically impossible to train due to lack of data. The same logic makes the multiclass classifier approach non-scalable for identifying even larger sets of diseases. For this reason, we took a different approach, which used a generic architecture that is separately trained for binary identification of each disease. Using this approach, given a set of *N* trained models and for any *m* < *N*, it takes *N* inference operations to detect a set of *m* existing disorders in a single ECG recording. Such network training is much more efficient, the results are more accurate, and, most importantly, requires a reasonable number of samples. Furthermore, with this approach, the effort required to extend the system to detect an additional disease is relatively low, simply requiring training of yet another model using the same architecture.

The advantages of our solution were demonstrated by binary classification (exist or not) of the eight most-common rhythm and/or morphological cardiac arrhythmias. The solution was also capable of identifying normal sinus rhythm. Each of the binary classification networks was trained independently, and therefore did not negatively impact other classes. Thus, these datasets comprising other disorders can be easily incorporated with existing training data and used with the same network architecture as developed here, without affecting the high accuracy of our system.

The deep network architecture constructed in this work was designed to be compatible with both digital 12-lead signals (i.e., 13 signals) and with standard plots of all leads, which are typically printed on paper. The presented generic architecture reached high accuracy for both CNN-ima and CNN-dig networks. Despite the increase in data dimensionality and the incorporation of redundant information, such as background pixels, CNN-ima suffered only a negligible reduction in accuracy. Our results show that the network can extract the important information from the images and ignore the redundant parts. In the age of smartphones and Internet of Things, images of an ECG plot can be acquired using a mobile device^24^. Doing so, however, is prone to background noise and change of plot perspective. The results of this work showed that our generic network architecture can cope with such input distortions without significantly affecting output accuracy.

The neural network approach is subject to inherent limitations, including reliance on large volumes of data. However, neural networks may offer a higher level of accuracy than other statistical methods as they are only dependent on the data and can operate independently of the biases of the investigator. We showed here that increasing the amount of data improved the AF detection accuracy. Moreover, the volume of data used here did not reach system accuracy saturation. Namely, additional data may very well further improve the results. This observation may also apply for the other tested disorders; it is plausible that our already highly accurate results relating to other disorders will further improve with the availability of additional datasets.

Because one-lead data can be measured from wearable devices, at first glance, it may seem appealing to develop a classification system based on single-lead data. Recent state-of-the-art classification type paper that is based on a single-lead ECG was presented by the Ng group^11^. Their study used a rather large dataset (91,232 single-lead ECG recordings collected from a total of 53,549 patients), but, because of the use of single-lead, they limited their diagnostics to a small set of diseases. Furthermore, because their classifier used a multiclass model, it is not clear how the presented work can be extended to other disorders. It also did not design to handle situations of more than one condition present in the same ECG. An additional state-of-the-art work was recently published by the Radinsky group^25^, which demonstrated application of deep neural networks for multiclass ECG classification. However, their database (MIT-DB^26^ from Physionet) included only 47 patients. Thus, the training and validation sets were not mutually exclusive with respect to patient identity, which could lead to potential overfitting.

A recent work attempted to automatically classify 12-lead ECG into 17 groups of conditions^27^. Although this work overcame the challenge of identifying both rhythm and morphology conditions and successfully detected more than one disease per ECG, it provided low accuracy and was only suitable for digital signals. Moreover, the database and the program are not public, rendering it impossible to compare its performance to ours. Note also that instead of standard 2.5-s 12-lead ECG reads, the work used 10 s recordings in each channel. A recent work using the same database to detect the same subset of cardiac diseases^28^, yielded results inferior to those reported here, in all categories and for accuracy in general (Table 4).

**Table 4:** Comparison of performance of the proposed algorithm versus other.

	Accuracy	
	State-of-art algorithm^28^ (based on Fig. 7)	Proposed method (for CNN-dig)
Normal	0.82	0.96
AF	0.92	0.98
I-AVB	0.78	0.99
LBBB	0.85	1.00
RBBB	0.96	0.99
PAC	0.74	0.92
PVC	0.87	0.92
STD	0.77	0.90
STE	0.50	0.98
Average accuracy	0.80	0.96

*AF* Atrial fibrillation, *I-AVB* first-degree atrioventricular block, *LBBB* left bundle branch block, *RBBB* right bundle branch block, *PAC* premature atrial contraction, *PVC* premature ventricular contraction, *STD* ST-segment depression, *STE* ST-segment elevation.

## Limitations versus advantages

This paper provides a proof of concept for the feasibility of automated detection of cardiac conditions using 12-lead ECG signals. The proposed system can use either standard digital or image of 12-lead ECG signals and is equally accurate with images containing smartphone camera acquisition artifacts.

One possible limitation of this work lay in the construction of an algorithm for rendering ECG images instead of using real images. The network may not be capable of dealing with real-world artifacts. A second possible limitation derived from use of a deep learning system instead of a known machine learning-based feature system. In machine learning systems, a set of features is designed, and the individual values of each feature can be analyzed. Because of the complex nature of neural networks, we are unable to indicate how different features of the ECG contributed to the final network output.

### Future works

One possibility for future work relates to the first limitation. We aim to develop open-source database of real ECG images (before and after camera scan) and test the algorithm performance. A second possible future work can relate to population homogeneity of the data. We aim to acquire 12-lead ECG standard digital or image from different machines and from different populations. A third possible future work should address the design of a model interpretability tool, to allow clinicians to understand which factors led to the AI system’s decision.

## Data Availability

The data analysis code, and a link to the datasets will be made freely available on GitHub following publication of the paper.
